# The GB Virus C (GBV-C) NS3 Serine Protease Inhibits HIV-1 Replication in a CD4+ T Lymphocyte Cell Line without Decreasing HIV Receptor Expression

**DOI:** 10.1371/journal.pone.0030653

**Published:** 2012-01-23

**Authors:** Sarah L. George, Dino Varmaz, John E. Tavis, Adnan Chowdhury

**Affiliations:** 1 Division of Infectious Diseases, Department of Internal Medicine, St. Louis University, St. Louis, Missouri, United States of America; 2 Research Service, St. Louis Veterans Affairs Medical Center, St. Louis University, St. Louis, Missouri, United States of America; 3 Department of Molecular Microbiology and Immunology, St. Louis University, St. Louis, Missouri, United States of America; Institut Pasteur, France

## Abstract

**Introduction:**

Persistent infection with GBV-C (GB Virus C), a non-pathogenic virus related to hepatitis C virus (HCV), prolongs survival in HIV infection. Two GBV-C proteins, NS5A and E2, have been shown previously to inhibit HIV replication *in vitro*. We investigated whether the GBV-C NS3 serine protease affects HIV replication.

**Results:**

GBV-C NS3 protease expressed in a human CD4+ T lymphocyte cell line significantly inhibited HIV replication. Addition of NS4A or NS4A/4B coding sequence to GBV-C NS3 increased the effect on HIV replication. Inhibition of HIV replication was dose-dependent and was not mediated by increased cell toxicity. Mutation of the NS3 catalytic serine to alanine resulted in loss of both HIV inhibition and protease activity. GBV-C NS3 expression did not measurably decrease CD4 or CXCR4 expression.

**Conclusion:**

GBV-C NS3 serine protease significantly inhibited HIV replication without decreasing HIV receptor expression. The requirement for an intact catalytic serine at the active site indicates that inhibition was mediated by proteolytic cleavage of an unidentified target(s).

## Introduction

Persistent infection with GB virus C (GBV-C) delays progression of HIV disease and significantly improves survival in HIV infection [1]–[5]. HIV/GBV-C coinfected people have delayed CD4+ T cell depletion, lower HIV viral loads, delayed progression to AIDS, and better response to HIV therapy than people infected with HIV alone [1]–[3], [5]–[13]. Untreated HIV-infected men with persistent GBV-C viremia, defined as GBV-C RNA detected in serum 5 or more years apart, have a 75% 10 year survival rate compared with 39% for untreated HIV-infected men without GBV-C viremia (p = 0.006) [2]. HIV-infected people with GBV-C viremia detected 2 or more years after HIV seroconversion have a 59% reduction in the risk of death compared with HIV-infected people who do not have GBV-C viremia [3]. GBV-C/HIV coinfected people treated with highly active anti-retroviral therapy (HAART) are significantly more likely to have a complete virologic response after initiation of therapy (46% vs. 28%, p = 0.036), and have a significantly larger median CD4+ T cell increase (240 cells/µL vs. 150 cells/µL, p = 0.05) [12]. GBV-C viremia is found in approximately 40% of HIV-infected people [1], [2], thus the effect of GBV-C on HIV disease potentially has a significant impact on outcomes in HIV-infected populations. GBV-C infection may also reduce the risk of HIV acquisition in some populations; maternal-to-child transmission of GBV-C reduces the risk of infant infection with HIV by 7-fold from 12% to 2% (p<0.001) [14].

**Figure 1:**
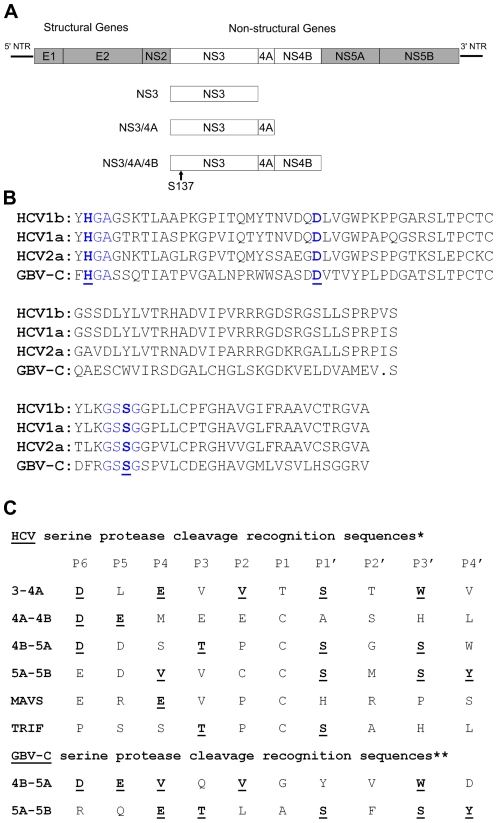
Schematic of the GBV-C genome and protein expression constructs. (**A**) The GBV-C genome, including the 5′ and 3′ non-translated regions (NTR), is shown. The structural genes include the envelope proteins E1 and E2, and the non-structural genes include NS2, NS3, NS4A, NS4B, NS5A, and NS5B. The regions coding for NS3, NS3/4A, and NS3/4A/4B that were cloned are shown. The relative position of the NS3 serine protease catalytic serine residue (S137) is shown. (**B**) Comparison of the HCV and GBV-C serine protease catalytic triad. Partial protease sequences of HCV1b (CON1 sequence), HCV1a (H77 sequence), HCV2a (JFH-1 sequence), are compared with the GBV-C AY196904 sequence. The catalytic triad consists of histidine 56, aspartic acid 79, and serine 137 (blue letters, underlined and in bold). Histidine 56 and serine 137 are expressed in similar peptide contexts in both HCV and GBV-C (blue letters). The GBV-C residues are numbered from the experimentally determined amino terminus of GBV-C NS3. (**C**) Comparison of the known cleavage recognition sequences of the HCV and GBV-C NS3 serine proteases. *Adapted from [42]. **Adapted from [17], [18].

GBV-C is a member of the family *Flaviviridae* and is the most closely related human virus to hepatitis C virus (HCV) [15]. Both GBV-C and HCV are single-strand positive sense RNA viruses whose proteins are translated as a single polyprotein (Fig. 1A); viral proteins are cleaved to their mature sizes by cellular and viral proteases [15], [16]. The largest protein in both the GBV-C and HCV open reading frames is non-structural protein 3 (NS3), which encodes a serine protease in the amino terminal end (Fig. 1B) and a helicase/ATPase in the carboxy terminal end [15], [17]–[21]. The protease catalytic residues histidine, aspartate, and serine are conserved between GBV-C and HCV (Fig. 1B) [17], however homology between the known cleavage substrates of GBV-C and HCV NS3 protease is limited (Fig. 1C). The NS3 protease of both GBV-C and HCV must be co-expressed with NS4A for efficient enzymatic activity [17], [20], [22], [23]. GBV-C NS3/4A mediates cleavage of NS3/NS4A, NS4B/NS5A, and NS5A/NS5B [17]. Cleavage is not detectable when the protease catalytic serine (residue 137) is mutated to alanine [17], similar to the proteases of HCV and other Flaviviruses [24], [25]. The residues of GBV-C NS4A which must be co-expressed with NS3 for efficient cleavage have been identified through deletion mapping [17]. The carboxy termini of NS3 and NS4A have not been experimentally determined, but the amino termini of NS3 and NS5A have been determined [17], thus the residues in the NS3/4A/4B polyprotein are known. The HCV NS3 protease cleaves a number of cellular proteins as well as the viral nonstructural proteins, including interferon mitochondrial antiviral signaling protein (MAVS) [26]–[28] and TRIF [29], altering intracellular signal transduction and initiation of a type I interferon response. The GBV-C NS3 protease has not been reported to cleave any non-GBV-C proteins.

The inhibitory mechanism of GBV-C infection on HIV replication has been partially characterized. In studies using whole virus from human serum or transfected viral RNA, coinfection of human peripheral blood mononuclear cells (PBMCs) and CD4+ T cells with GBV-C significantly inhibits HIV replication *in vitro* [1], [30], [31]. GBV-C infection of PBMCs and CD4+ T cells increases secretion of CCR5 ligand chemokines and decreases CCR5 expression by 50% compared with mock-infected PBMCs [30], [31]. Two GBV-C proteins, NS5A [32]–[35] and the envelope glycoprotein E2 [36]–[40], inhibit HIV replication in CD4+ Jurkat cells by either decreasing HIV receptor expression (NS5A) or blocking HIV attachment and entry (E2). NS5A expressed in CD4+ Jurkat T cells inhibits HIV replication by decreasing expression of CD4 [35] and CXCR4 [32]–[34]. This effect is mediated in part by enhanced secretion of the CXCR4 ligand chemokine SDF-1 [33], [34]. GBV-C peptides derived from the E2 protein bind to HIV gp41 fusion peptide, blocking HIV binding [37], [38], [40]. Anti-GBV-C E2 antibodies neutralize infectious HIV particles and HIV gag particles, however they do not inhibit entry of HIV pseudotyped VSV particles [39]. Anti-E2 antibodies interfere with HIV attachment to cell membranes but do not block HIV entry to cells [39]. The authors propose that anti-E2 antibodies neutralize HIV by interacting with a cellular antigen on HIV particles [39]. In addition to these characterized HIV inhibitory mechanisms, GBV-C infection *in vivo* decreases T cell activation independently of HIV viral load [41], though the mechanism is unknown.

Here, we explored whether expression of GBV-C NS3 in a CD4+ T lymphocyte cell line affects HIV replication. Since the homologous HCV protease cleaves multiple viral and cellular proteins (Fig. 1C) [17], [42], we hypothesized that GBV-C protease may perturb the cellular environment sufficiently to affect HIV replication.

## Results

**Figure 2:**
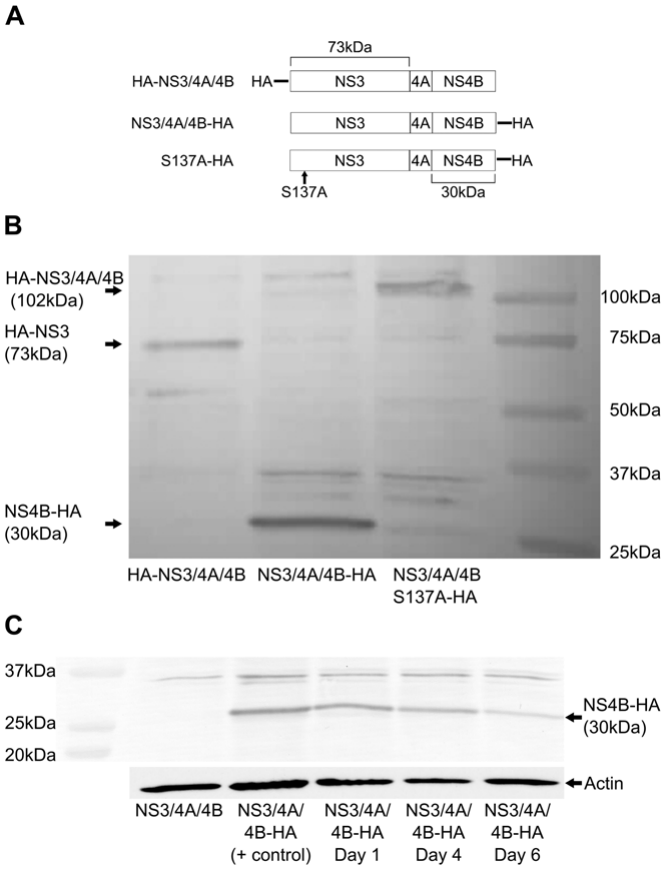
GBV-C NS3/4A/4B expression and protease activity in Jurkat cells. (**A**) Relative primary structures of the GBV-C proteins employed are shown. HA indicates the location of the HA epitope tag. The location of serine 137 is indicated along with the relative sizes of NS3 and NS4B. (**B**) CD4+ Jurkat cells were transfected with equal amounts of HA-NS3/4A/4B, NS3/4A/4B-HA, or S137A-HA expression plasmids and cell lysates were collected one day post transfection. Cell lysates were analyzed by western blot developed with an anti-HA monoclonal antibody. Molecular weight markers are shown at right, and the mobility of NS3 (73kDa), NS4B (30kDa), or the complete polypeptide S137A (102kDa) is shown at left. (**C**) Jurkat cells were transfected with NS3/4A/4B-HA and equal numbers of viable cells determined by trypan blue staining were lysed on days 1, 4, and 6 post transfection. Protein from lysates was analyzed by western blots developed with an anti-HA monoclonal antibody. Expression of GBV-C NS3/4A/4B-HA on days 1, 4, and 6 post transfection is shown compared with cell lysates from cells transfected with NS3/4A/4B and a highly concentrated NS3/4A/4B-HA cell lysate. Molecular weight markers are indicated at the left, and the mobility of NS4B-HA (30kDa) is indicated at the right. A western blot of actin from the same cell lysates run in parallel is shown below as a loading control.

We first determined whether GBV-C NS3 is a functional protease when expressed in CD4+ Jurkat cells. Jurkats were chosen as host cells to approximate the natural CD4+ T lymphocyte host cell of both GBV-C [43] and HIV. The coding sequences for NS3, NS3/4A, and NS3/4A/4B were separately amplified from a full-length GBV-C genome (GenBank AY196904) [44] and inserted into pCDNA3.1/Zeo+ (Fig. 1A). The carboxy termini of NS3 and NS4A had to be approximated as they have not been experimentally determined [17]. To measure proteolytic cleavage of the NS3/4A/4B polyprotein, we inserted HA-tags on the amino- and carboxy-termini of NS3/4A/4B (Fig. 2A). To determine if detectable proteolytic cleavage would be lost with mutation of the catalytic serine, as described previously [17], we mutated serine 137 to alanine, creating the carboxy terminally HA-tagged construct S137A (Fig. 2A).

As shown in Figure 2B, a 73kDa band corresponding to the predicted size of HA-NS3 was seen in lysates of cells transfected with HA-NS3/4A/4B, and a 30kDa band corresponding to the predicted size of NS4B-HA was seen in lysates of cells transfected with NS3/4A/4B-HA. A 102kDa band corresponding to the predicted size of full-length NS3/4A/4B was seen in lysates of cells transfected with S137A-HA. Lysates of cells transfected with S137A-HA did not show a either a 73kDa band corresponding to NS3 or a 30kDa band corresponding to NS4B, indicating proteolytic activity was lost with mutation of the catalytic serine. An uncleaved 102kDa band was not seen in cells transfected with HA-NS3/4A/4B or NS3/4A/4B-HA, indicating proteolytic cleavage was sufficiently rapid to prevent detectable accumulation of the precursor polyprotein. To evaluate the longevity of protein expression in this system, we transfected cells with NS3/4A/4B-HA and measured protein expression for six days by western analysis (Fig. 2C). Equal numbers of viable cells were harvested on days 1, 3, and 6 post transfection and lysed in the same volume of buffer. The same volume of cell lysate was separated on SDS-PAGE gels and GBV-C protein expression was measured in parallel (Fig. 2C). GBV-C protein expression remained essentially unchanged between days 1 and 3 and was still detectable though diminished on day 6.

**Figure 3:**
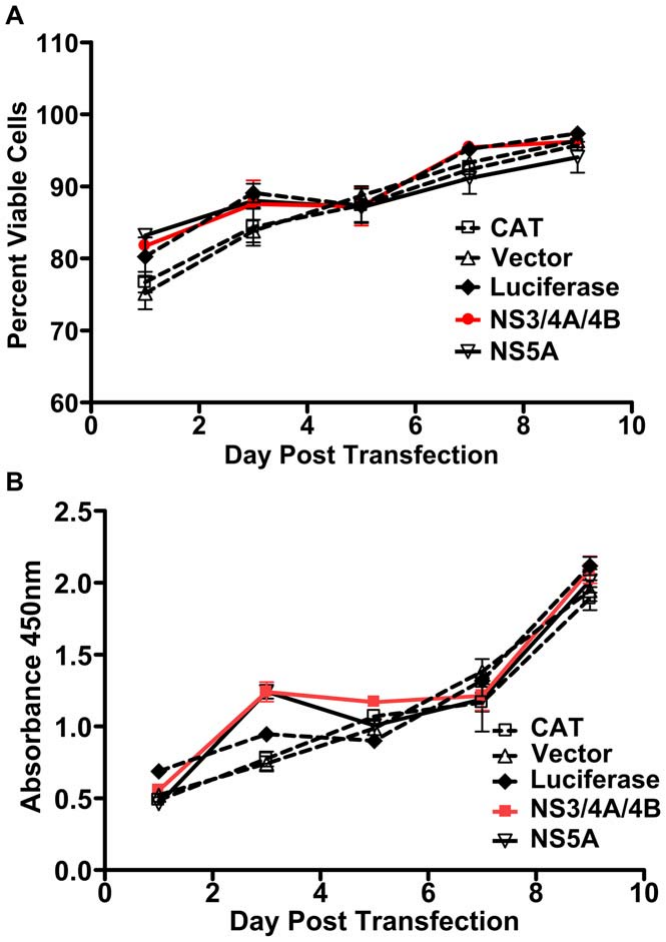
Transfection of Jurkat cells with GBV-C NS3/4A/4B or NS5A expression plasmids does not cause cellular toxicity. Jurkat cells were transfected with equal amounts of NS3/4A/4B (red line), NS5A (solid black line), or control protein (CAT, luciferase) expression plasmids in pCDNA3.1/Zeo+ or empty vector (dotted black lines). Cellular viability (**A**) and proliferation (**B**) were measured for 10 days. Standard errors for each measurement on each day are shown.

To determine whether expression of GBV-C NS3/4A/4B caused significant cellular toxicity, we compared viability and proliferation of Jurkat cells transfected with plasmids expressing GBV-C NS3/4A/4B, GBV-C NS5A (amplified from GBV-C clone AY196904 [44]), chloramphenicol acetyl transferase (CAT), luciferase, or empty vector for up to 10 days. All proteins were expressed from pCDNA3.1/Zeo+. CAT and luciferase expression plasmids were used as irrelevant protein controls. Expression of both CAT and luciferase in transfected cells was confirmed by CAT ELISAs and luciferase assays (data not shown). As shown in Figures 3A and B, there was no difference in viability or proliferation of cells transfected with GBV-C or control protein expression plasmids for up to 10 days after transfection.

To investigate whether GBV-C NS3 expression affected HIV replication, we transfected Jurkat cells with plasmids expressing either NS3, NS3/4A, NS3/4A/4B, CAT, glutathione-s-transferase (GST), or empty vector plus the HIV pNL4-3 genomic expression plasmid [45]. NS3 expressed without NS4A would be expected to have less efficient protease activity than NS3 expressed with NS4A based on homology with HCV [20], [22], [23]. As the carboxy terminus of NS4A has not been experimentally determined, we transfected cells with NS3/4A/4B to ensure we were expressing the full-length NS4A protein. HIV pNL4-3 plasmid was chosen as the source of replication-competent HIV to ensure efficient HIV replication during the time the transfected GBV-C or control proteins were maximally expressed (Fig. 2C).

**Figure 4:**
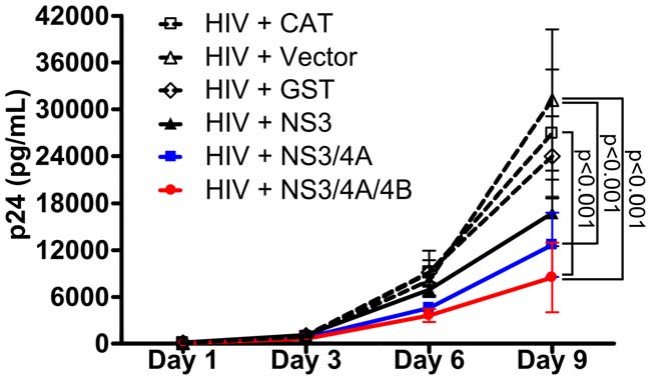
Expression of GBV-C NS3, NS3/4A, or NS3/4A/4B in Jurkat cells inhibits HIV replication for up to 9 days. Jurkat cells were transfected with equal amounts of plasmids expressing NS3 (solid black line), NS3/4A (blue line), NS3/4A/4B (red line), GST, CAT, or empty vector (dotted black lines) plus HIV pNL4-3 plasmid. Mean and standard error p24 values of supernatants collected on days 1, 3, 6, and 9 are shown.

As shown in Figure 4, expression of both NS3/4A and NS3/4A/4B significantly inhibited HIV replication through day 9, while NS3 expressed alone was less inhibitory. NS3/4A/4B significantly inhibited HIV replication compared with CAT (p<0.001), empty vector (p<0.001), and GST (p<0.01) on day 9. NS3/4A significantly inhibited HIV compared with CAT (p<0.01) and vector (p<0.001) on day 9. NS3/4A inhibited HIV compared with GST on day 9, but the difference was not statistically significant. While a difference in HIV replication between cells transfected with NS3 compared with CAT, GST, or empty vector was apparent, the difference was not statistically significant. Expression of NS3/4A/4B inhibited HIV replication starting on day 3 post-transfection and continued through day 9. Expression of NS3/4A or NS3 inhibited HIV replication by day 6 post-transfection. While there was a trend to stronger inhibition by expression of NS3/4A/4B compared with NS3/4A, the difference was not statistically significant.

Therefore, expression of GBV-C NS3, NS3/4A, and NS3/4A/4B inhibited HIV replication in Jurkat cells compared with cells transfected with two irrelevant control proteins. Addition of NS4A coding sequence to NS3 increased the inhibitory effect on HIV replication, potentially pointing to a role for NS3 enzymatic activity in inhibition of HIV [17], [20], [22], [23]. Since expression of NS3/4A/4B was somewhat more inhibitory for HIV replication than expression of NS3/4A, and both plasmids had equal effects on cellular viability and proliferation (data not shown), either: 1) NS4B expression had a separate inhibitory effect on HIV replication, 2) NS4B enhanced the stability, localization, or enzymatic activity of NS3/4A [46], or 3) our NS3/4A construct did not contain the full-length NS4A sequence, and thus its enzymatic activity and inhibitory effect was reduced relative to NS3/4A/4B.

**Figure 5:**
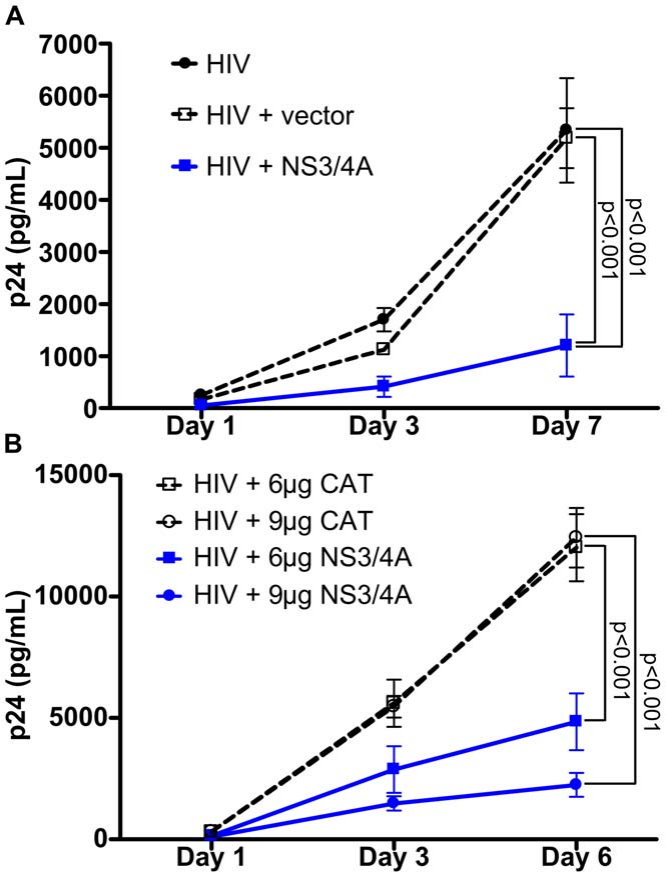
GBV-C NS3/4A inhibition of HIV replication in Jurkat cells is dose-dependent. (**A**) Jurkat cells were transfected with either 3 µg HIV pNL4-3 (HIV), 3 µg of HIV pNL4-3+3 µg pCDNA3.1/Zeo+ (HIV+vector), or 3 µg HIV pNL4-3+3 µg NS3/4A in pCDNA3.1/Zeo+ (HIV+NS3/4A) (blue line). Mean and standard errors of p24 values in supernatants collected on days 1, 3, and 7 are shown. (**B**) Jurkat cells were transfected with HIV pNL4-3 and either 6 or 9 µg of NS3/4A expression plasmid (blue lines) or equivalent amounts of CAT expression plasmid; mean and standard errors of p24 values in supernatants collected on days 1, 3, and 6 are shown.

To confirm that expression of NS3/4A alone could inhibit HIV replication in Jurkat cells, we transfected cells with the expression plasmid for NS3/4A or empty vector plus HIV pNL4-3 plasmid or HIV pNL4-3 plasmid alone. GBV-C NS3/4A expression significantly inhibited accumulation of p24 in supernatants (p<0.001 on day 7), while equivalent amounts of empty vector did not (Fig. 5A). To determine if NS3/4A's inhibitory effect on HIV was dose-dependent, we transfected Jurkat cells with 6 or 9 µg NS3/4A expression plasmid or equal amounts of CAT expression plasmid plus HIV pNL4-3 plasmid. As shown in Figure 5B, 6 µg of NS3/4A expression plasmid significantly inhibited HIV replication compared with equivalent amounts of CAT expression plasmid on day 6 (p<0.001). 9 µg of NS3/4A expression plasmid significantly inhibited HIV replication compared with equivalent amounts of CAT expression plasmid on days 3 (p<0.01) and 6 (p<0.001). Thus, increasing the dose of NS3/4A expression plasmid increased the inhibitory effect on HIV replication.

**Figure 6:**
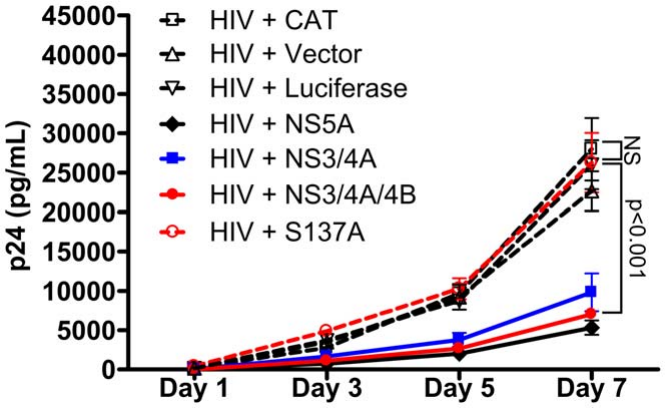
Mutating the protease catalytic serine of GBV-C NS3/4A/4B to alanine results in loss of HIV inhibition. Jurkat cells were transfected with HIV pNL4-3 and equal amounts of NS3/4A (blue line), NS3/4A/4B (red line), NS5A (solid black line), S137A (dotted red line), CAT, or luciferase expression plasmids or empty vector (dotted black lines). Supernatants were collected and p24 measured on days 1, 3, 5, and 7. Mean and standard error of p24 values are shown.

Since addition of NS4A or NS4A/4B coding sequences to NS3 substantially increased its inhibitory effect on HIV replication and thus implicated NS3 enzymatic activity in the mechanism of inhibition (Fig. 4), we investigated whether mutation of the NS3 protease catalytic serine 137 to alanine would result in loss of the HIV inhibitory effect. Since our western blot data demonstrated that mutation of serine 137 to alanine (S137A) resulted in loss of detectable protease cleavage products (Fig. 2B), we compared inhibition of HIV replication in cells transfected with either NS3/4A, NS3/4A/4B, S137A, NS5A, CAT, or luciferase expression plasmids or vector plus HIV pNL4-3 (Fig. 6). NS5A expression was a positive control for inhibition of HIV replication [32]-[35]. There was no significant difference in p24 values between supernatants of cells transfected with S137A, CAT, or luciferase expression plasmids or empty vector (Fig. 6). There was also no significant difference in p24 values between supernatants of cells transfected with NS3/4A, NS3/4A/4B or NS5A. There was a trend toward stronger inhibition by NS3/4A/4B and NS5A compared with NS3/4A but the difference was not statistically significant. NS3/4A, NS3/4A/4B, and NS5A significantly inhibited HIV replication compared with S137A, CAT, and luciferase expression plasmids and empty vector on day 7 (p<0.001). NS3/4A/4B and NS5A significantly inhibited HIV replication compared with S137A, CAT, and luciferase expression plasmids and empty vector on day 5 (CAT and S137A, p<0.01; luciferase and vector, p<0.05), while NS3/4A significantly inhibited HIV replication compared with S137A and CAT on day 5 (S137A, p<0.01; CAT, p<0.05). Therefore, mutating the protease catalytic serine to alanine resulted in loss of both measurable proteolytic activity (Fig. 2B) and inhibition of HIV replication (Fig. 6).

As GBV-C NS5A expression decreases CD4 and CXCR4 expression in Jurkat cells *in vitro* [32]–[35], we investigated whether GBV-C NS3/4A/4B decreased HIV receptor expression in transfected Jurkat cells. The NS3/4A/4B construct was chosen as it expresses NS3, NS4A, and NS4B and inhibited HIV replication as much as NS5A expression in this system (Fig. 6). Since no antibodies are available which reliably detect GBV-C NS3, NS4A, or NS4B expression, and we wished to avoid the use of an HA tag and express only the native proteins, Jurkat cells were transfected with 1 µg green fluorescent protein (GFP) in pCDNA3.1/Zeo+ as a marker of protein expression after transfection plus either 6 µg NS3/4A/4B or CAT expression plasmids and a scrambled human CD4 siRNA control [47], [48]. As a positive control to demonstrate suppression of a cell surface marker on these cells, we co-transfected cells with anti-human CD4 siRNA plus 1 µg GFP and 6 µg CAT expression plasmid [47], [48].

**Figure 7:**
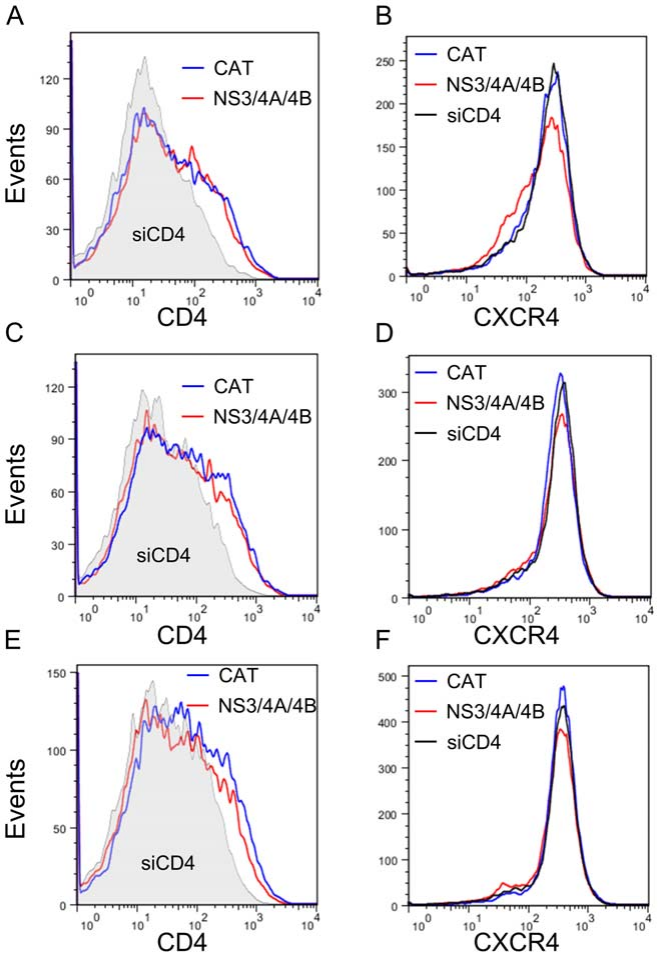
GBV-C NS3/4A/4B expression does not decrease CD4 or CXCR4 expression. Jurkat cells were transfected with 1 µg GFP in pCDNA3.1/Zeo+ and either 6 µg NS3/4A/4B expression plasmid (red line) or CAT expression plasmid (blue line) and scrambled anti-CD4 siRNA. Equal numbers of viable cells were labeled with anti-CD4 PE-Cy7 and anti-CXCR4-APC and analyzed by FACS on days 3 (**A** and **B**), 4 (**C** and **D**), and 5 (**E** and **F**) post transfection. Cells were gated for GFP expression and then analyzed for CD4 and CXCR4 expression. As a control to demonstrate reduction in receptor expression, cells were transfected with 1 µg GFP in pCDNA3.1/Zeo+ and 6 µg CAT expression plasmid plus anti-CD4 siRNA (**A,C,E**: gray shaded area, **B,D,F**: black line).

We compared CD4 and CXCR4 expression on Jurkat cells transfected with NS3/4A/4B or CAT expression plasmids on days 3, 4, and 5 after transfection. These days were chosen based on preliminary experiments identifying the time required to demonstrate CD4 knockdown in CD4 siRNA transfected cells. Equal numbers of viable cells were collected on each day and gated for GFP expression by comparison with cells transfected with CAT expression plasmid and scrambled CD4 siRNA but no GFP. GFP+ cells were analyzed for CD4 and CXCR4 expression. The percent of cells expressing GFP ranged from 39–65% on day 3, 36–57% on day 4, and 34–51% on day 5 (data not shown). There was no difference in the percentage of cells expressing GFP in cells transfected with NS3/4A/4B compared with CAT expression plasmids. As shown in Figures 7A and C, there was no difference in CD4 expression in cells transfected with NS3/4A/4B compared with CAT expression plasmids on days 3 and 4. There was a trend to a slight decrease in CD4 expression in cells transfected with NS3/4A/4B compared with CAT expression plasmids on day 5, but peak expression was not significantly shifted (Fig. 7E). There was a significant decrease in the mean fluorescent intensity (MFI) of CD4 expression in cells transfected with CD4 siRNA compared with cells transfected with scrambled CD4 siRNA and CAT or NS3/4A/4B expression plasmids on days 3, 4, and 5 (p<0.0001) (Figs. 7A, C, E). There was no decrease in CXCR4 expression in cells transfected with NS3/4A/4B compared with CAT expression plasmids on days 4 and 5 (Figs. 7D and F). There was a slight decrease in CXCR4 expression in cells transfected with NS3/4A/4B compared with CAT expression plasmids on day 3 (Fig. 7B). There was no decrease in CXCR4 expression in cells transfected with CD4 siRNA compared with cells transfected with scrambled CD4 siRNA and NS3/4A/4B or CAT expression plasmids, as expected (Figs. 7B, D, F). Therefore, we were unable to find evidence of significant, sustained decreased expression of either CD4 or CXCR4 in cells transfected with NS3/4A/4B compared with CAT expression plasmids on days 3, 4, or 5 after transfection. Since inhibition of HIV replication is seen by 3 days after transfection of GBV-C expression plasmids (Figs. 4, 5A and B, and Fig. 6) and is maintained for up to 9 days (Fig. 4), the NS3 protease inhibits HIV replication by a mechanism other than decreasing HIV receptor expression.

## Discussion

We have shown that transient expression of GBV-C NS3 protease in the human Jurkat CD4+ T cell line significantly inhibited HIV replication, and that inhibition required an intact protease catalytic serine (Fig. 6). Mutation of the catalytic serine 137 to alanine resulted in loss of both detectable protease cleavage products (Fig. 2B) and loss of inhibition of HIV replication (Fig. 6). While NS3 expressed alone was inhibitory (Fig. 4), addition of NS4A or NS4A/NS4B coding sequences substantially increased the inhibitory effect (Fig. 4). These data, combined with the evidence that an intact catalytic serine was required for inhibition, provide evidence that inhibition of HIV replication by the GBV-C NS3 protease was mediated by proteolytic cleavage. What the cleavage target(s) of the protease are remains unknown. Potential targets include cleavage of HIV proteins or cleavage of cellular proteins which HIV requires for efficient replication.

Addition of NS4B coding sequences to NS3/4A consistently increased the inhibitory effect on HIV replication, though the difference was not significant (Figs. 4 and 6). Therefore, NS4B expression may have a modest additional inhibitory effect on HIV replication independent of NS3/4A. In HCV, NS4B mediates formation of the membranous web, a region of modified cellular membranes upon which viral replication occurs [49], [50]. Whether GBV-C NS4B has similar functions is unknown, but given the homology between GBV-C and HCV, it is plausible that GBV-C NS4B may disrupt intracellular membranes similar to HCV NS4B, and this could interfere with efficient HIV replication if both viruses infect the same CD4+ T cell. It is unknown whether GBV-C and HIV coinfect the same cells *in vivo* or *in vitro*. Whether GBV-C NS4B has HIV inhibitory effects will need to be defined in future work.

GBV-C NS3/4A/4B expression did not significantly decrease CD4 or CXCR4 expression on Jurkat cells on days 3, 4, or 5 post-transfection (Fig. 7). As inhibition of HIV replication was seen beginning day 3 and lasting through day 9 post transfection, the GBV-C protease likely inhibited HIV replication by a mechanism which does not require decreased HIV receptor expression. This would be a novel mechanism for GBV-C-mediated inhibition of HIV.

We found transfected Jurkats were poorly permissive for HIV infection up to 48 hours after transfection using high-titer supernatants harvested from pNL4-3 transfected cells which were infectable into jurkats which had not been transfected (data not shown). This was seen regardless of the plasmid transfected (GBV-C or control expression plasmid), so was likely due to nonspecific cellular effects of the electroporation. This argues that most of the p24 antigen in our culture supernatants at least through day 3 was probably translated from mRNA transcribed from the HIV DNA derived from the pNL4-3 plasmid, and not from a complete viral infection cycle. Since p24 levels were reduced in supernatants of cells transfected with GBV-C expression plasmids as early as day 3 post transfection (Fig. 4, Figs. 5A and B, Fig. 6), we hypothesize that GBV-C serine protease expression may inhibit HIV at a step after HIV DNA integration, such as mRNA transcription, processing, translation, or virion assembly. This is in contrast to GBV-C NS5A and E2 proteins, both of which block HIV replication at the attachment/entry/fusion steps [32]–[40].

In summary, we have identified a novel HIV-inhibitory GBV-C protein, the NS3 protease, which requires an intact catalytic serine to inhibit HIV replication. In this *in vitr*o system, inhibition of HIV replication by GBV-C protease was similar in strength to inhibition by the previously-described GBV-C NS5A protein [32]–[35]. Expression of NS3 protease did not decrease HIV receptor expression. The mechanism by which GBV-C NS3 protease inhibits HIV replication, and the possible role of NS4B in inhibition of HIV replication, will need to be defined in future work.

## Materials and Methods

### Synthesis of GBV-C expression vectors

The coding regions of GBV-C NS3, NS3/4A, NS3/4A/4B, and NS5A were amplified from the full-length GBV-C AY196904 sequence [44] and inserted into pCDNA3.1/Zeo+ expression vector (Invitrogen). The coding sequence for NS3 was nt 2682–4556, NS3/4A was nt 2682–4727, NS3/4A/4B was nt 2682–5600, and NS5A was nt 5601–6938 (numbered from the methionine codon at the beginning of E1). The carboxy termini of NS3 and NS4A have not been experimentally defined, so the GBV-C NS4A sequence identified as the required cofactor for activity of the GBV-C NS3 protease was used as the approximate carboxy terminus of NS3/4A [17]. To avoid including any NS4A residues in the NS3 construct, the carboxy terminus of NS3 was placed ten amino acids upstream of the start of the NS4A cofactor sequence. The NS5A sequence had the same amino and carboxy termini as the sequence studied by Stapleton et al [32]–[35], however it was amplified from a different source GBV-C sequence (AY196904) [44]. The S137A construct was created from the NS3/4A/4B sequence by mutating the thymidine residue at position 3090 to guanine. HA tags were inserted on the amino and carboxy termini of NS3/4A/4B and the carboxy terminus of S137A (Fig. 2A) using PCR. Sequences were amplified using Roche HiFi DNA polymerase and inserts were completely sequenced.

### Control expression plasmids

Chloramphenicol acetyl transferase (CAT) [9], glutathione-s-transferase (GST), and luciferase [51], all under the control of the CMV promoter in pCDNA3.1/Zeo+ vector, were used as irrelevant protein expression controls. Plasmid DNAs for transfection were prepared with endotoxin-free maxipreps (Qiagen). HIV pNL4-3 plasmid was obtained from the NIH AIDS Reagent program [45].

### Cells

CD4+ CXCR4+ Jurkat cells [52] were maintained in RPMI supplemented with 10% fetal calf serum and 1% penicillin/streptomycin/L-glutamine.

### Western blots

5×10^6^ Jurkat cells were transfected in triplicate with 6 µg GBV-C expression or control plasmids using an Amaxa II nucleofection device, according to the manufacturer's instructions. Transfected cells from the triplicate transfections were combined and harvested on day 1 (Fig. 2B), or duplicate transfections were combined and lysed on days 1, 3, and 6 (Fig. 2C). Lysis was performed in RIPA buffer supplemented with protease inhibitor (Sigma), and protein content measured using BCA assay (Pierce). Cell lysates were loaded onto 4–20% SDS-PAGE gels, transferred to PVDF membranes, and western blots were developed with anti-HA high affinity monoclonal antibody (Roche 3F-10).

### Viability and proliferation assays

5×10^6^ Jurkat cells were transfected with 6 µg GBV-C expression or control plasmids in triplicate. Viability was measured by trypan blue staining and proliferation was measured using BrdU incorporation assays (Roche). For trypan blue staining, aliquots of cells were mixed with equal amounts of trypan blue and the number of viable cells was determined on a Cedex cell counter (Roche). For BrdU assays, cells were counted on day 1 post-transfection and each transfection was plated in duplicate in ten 96 well plates (20,000 cells/well) to provide one plate per timepoint per assay. Plates were assayed for viability and proliferation for 10 days post-transfection. BrdU was added 24 hours prior to cell lysis to allow sufficient time for DNA incorporation. After 24 hours, cells were fixed, stained with anti-BrdU POD antibody (Roche) and absorbance of BrdU was read at 440 nm.

### HIV transfections

5×10^6^ Jurkat cells were transfected with 6 µg (unless otherwise specified) GBV-C or control expression plasmid plus 1 µg (unless otherwise indicated) HIV pNL4-3 plasmid in triplicate. Transfections were performed in an Amaxa Nucleofector II using Nucleofection kit V. After transfection, cells were incubated in RPMI supplemented with 10% fetal calf serum and 1% penicillin/streptomycin/L-glutamine. Cell-free supernatants were collected on the days indicated by centrifuging 650 µL of supernatant at 300 x *g* for 5 minutes removing the supernatant for analysis. Supernatants were kept frozen at −80°C until p24 determination in p24 ELISAs (Zeptometrix) according to the manufacturer's instructions. The p24 standard curve was derived by linear regression using Excel software and used to calculate the p24 content of cell supernatants.

### HIV receptor expression

5×10^6^ Jurkat cells were transfected with 1 µg GFP expression sequence cloned into pCDNA3.1/Zeo+ (a marker for successful transfection and protein expression) together with 6 µg NS3/4A/4B or CAT expression plasmids and 500 pmole scrambled anti-human CD4 siRNA [47], [48]. As a control for measuring receptor knock-down, cells were transfected with 1 µg GFP in pCDNA3.1/Zeo+ with 6 µg CAT expression vector and 500 pmole anti-human CD4 siRNA (for a final concentration of 5 µM in the 100 µL transfection buffer) [47], [48]. Equal numbers of viable cells were harvested on days 3, 4, and 5 and stained with anti-CD4 PE-Cy7 and anti-CXCR4-PE (BD Pharmingen) and analyzed by flow activated cell sorting (FACS) on a BD LSR II Flow Cytometer [30]. Cells were analyzed first for GFP expression in comparison with a no GFP control (CAT expression plasmid plus scrambled anti-CD4 siRNA), then for CD4 and CXCR4 expression. At least 10,000 gated events were analyzed for each condition on each day.

### Statistics

Two-way ANOVAs were used to compare the means of p24 values from supernatants collected across multiple days, with Bonferroni's post-test comparison to compare individual means. Standard errors of means were calculated using Excel or GraphPad Prism software.
